# Impact of *MTHFR C677T* polymorphism on recurrent spontaneous abortion: Bridging hyperhomocysteinemia, bile acid metabolism, and the epigenetic–immune axis

**DOI:** 10.1016/j.gendis.2026.102293

**Published:** 2026-06-10

**Authors:** Na Yu, Yuxian Wu, Ting Sun, Yang Liu, Yaoyang Liu, Shihua Bao

**Affiliations:** a Department of Reproductive Immunology, Shanghai Key Laboratory of Maternal-Fetal Medicine, Shanghai Institute of Maternal-Fetal Medicine and Gynecologic Oncology, Shanghai First Maternity and Infant Hospital, School of Medicine, Tongji University, Shanghai 200092, China; b Department of Biological Therapy, The Third Affiliated Hospital, Naval Medical University, Shanghai 200082, China; c Department of Critical Care Medicine, Naval Medical Center of PLA, Naval Medical University, Shanghai 200052, China; d School of Medicine, Shanghai University, Shanghai 200940, China; e Department of Clinical Genetics, The Second Affiliated Hospital, Naval Medical University, Shanghai 200001, China

*MTHFR* C677T is a recognized genetic risk factor for recurrent spontaneous abortion (RSA), classically mediated through hyperhomocysteinemia (HHcy).^1^ However, HHcy alone does not fully explain clinical heterogeneity. We performed multi-omics profiling of 196 RSA patients stratified by *MTHFR* C677T genotype (CC/CT/TT = 66/64/66), revealing that the *MTHFR* 677 TT genotype defines a distinct subgroup characterized by a clinical pattern of elevated estrogen, reduced androgens, and HHcy. Beyond this, *MTHFR* 677 TT carriers exhibited a pronounced Th17/Th1-skewed immune response, widespread downregulation of serum bile acids, and genome-wide promoter hypomethylation. Our integrated analysis establishes that the *MTHFR* 677 TT genotype drives pathogenic immune deviation via the combined effects of HHcy, bile acid dysregulation, and DNA hypomethylation. These findings provide a novel rationale for targeted management, such as folate combined with bile acid modulation, in this RSA subgroup.

This cross-sectional study enrolled 196 RSA patients grouped by *MTHFR* C677T genotype (Fig. 1A). Data collected included demographics, clinical parameters, peripheral lymphocyte subsets and cytokines, serum metabolomics (UPLC-MS), and genome-wide DNA methylation (RRBS). The baseline clinical characteristics and comparisons between patients with TT genotype and those with *MTHFR* 677CC/CT genotype are presented in Table S1.1-1.7. Compared to the *MTHFR* 677CC/CT groups, *MTHFR* 677 TT carriers showed significantly higher homocysteine (HCY) and estrogen, and lower androgen levels, confirming a distinct clinical-metabolic-endocrine triad (Fig. 1B, upper panel). Autoantibody positivity rates did not differ across genotypes (Table S1.7), though the autoantibody profile distribution (Fig. S4) indicated distinct top-ranked specificities.

**Figure 1:**
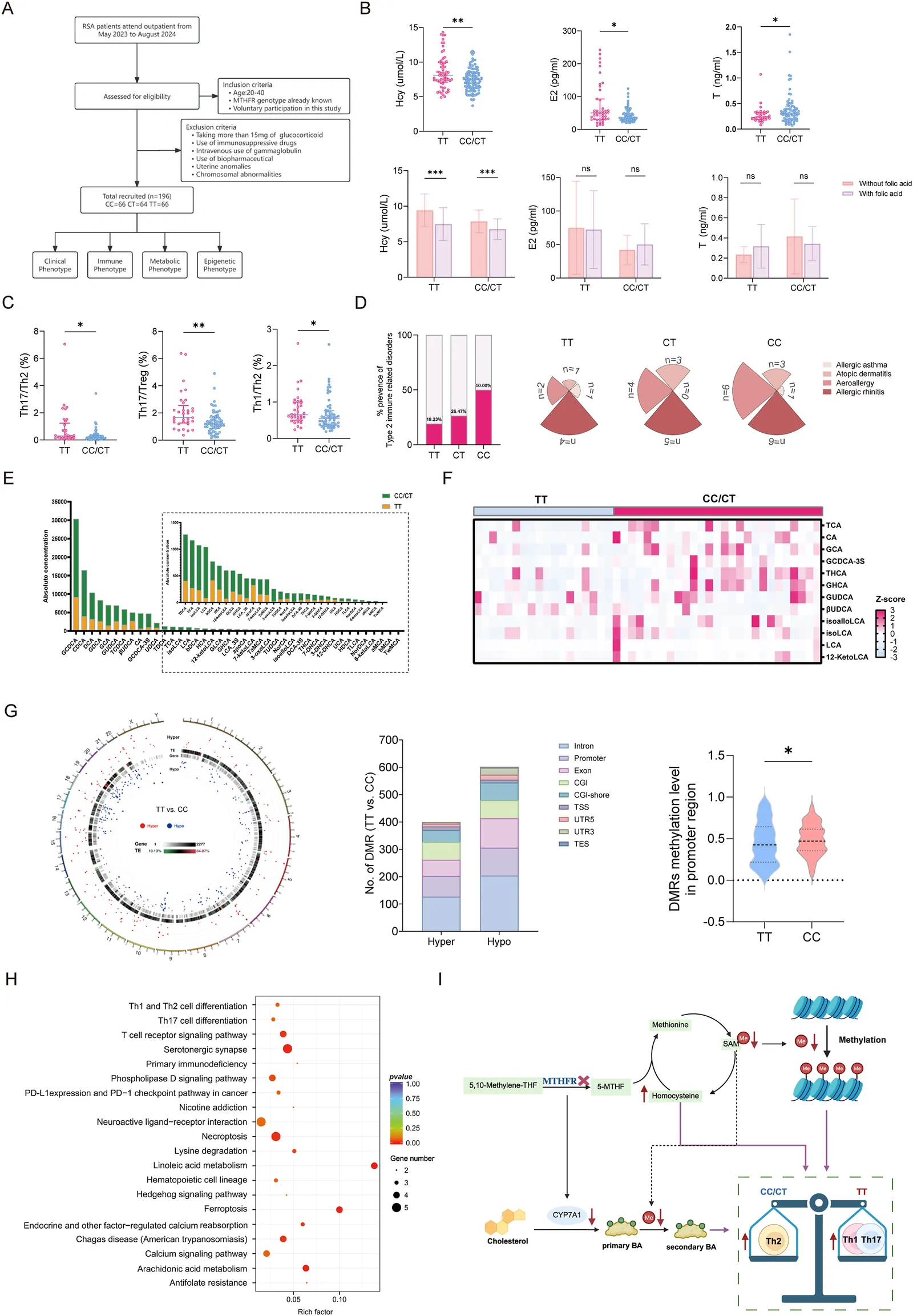
Clinical characteristics and multi-omics analysis of RSA patients stratified by *MTHFR* C677T genotypes. **(A)** Flow diagram illustrating the recruitment process, inclusion/exclusion criteria, and study cohort classification of women with RSA. **(B)** Serum marker comparisons. Upper panel: Plasma levels of Hcy, E2, and T in RSA patients carrying the *MTHFR* 677 TT genotype versus those with the 677CC/CT genotypes. Lower panel: Levels of Hcy, E2, and T in patients receiving folic acid supplementation versus those without supplementation, analyzed across genotypes. **(C)** Immune cell balance across *MTHFR* C677T genotypes. Scatter plots show the ratios of Th17/Treg, Th17/Th2, and Th1/Th2 cell populations among individuals with different *MTHFR* C677T genotypes. **(D)** Association of *MTHFR* genotypes with Type 2 immunity-related disorders. Left: Prevalence of atopic dermatitis, aeroallergy, allergic rhinitis, and allergic asthma across *MTHFR* genotype groups. Right: Composition of these Type 2 disorders within each genotype, illustrated by pie charts. **(E)** Stacked bar chart of absolute serum concentrations of 41 bile acid metabolites in women with RSA, stratified by *MTHFR* 677 TT *vs*. 677CC/CT genotypes. **(F)** Heatmap depicting bile acid metabolites that are differentially abundant between the two genotype groups in RSA patients. Color intensity represents Z-scores, with blue indicating decreased abundance (down to −3) and red indicating increased abundance (up to +3). **(G)** Influence of *MTHFR* C677T genotype on the epigenetic landscape in RSA. Left: Genome-wide distribution of differentially methylated CpGs between the *MTHFR* 677 TT and 677CC groups. Chromosome ideograms are shown in the outer ring; inner arcs represent DMR density. Hypermethylated and hypomethylated sites are highlighted in red and blue, respectively. Middle: Stacked bar chart showing the genomic distribution (Promoter, Intron, Exon, etc.) of hyper- and hypomethylated DMRs in the TT versus CC comparison. Right: Methylation levels of promoter-associated DMRs in the two genotype groups, presented as violin plots with embedded boxplots showing medians and interquartile ranges. **(H)** Bubble plot showing the top 20 enriched KEGG pathways for genes associated with DMRs. Dot size represents the number of genes mapped to each pathway; the *x*-axis indicates the enrichment ratio (rich factor), and color intensity reflects the statistical significance (−log_10_-transformed *p*-value). **(I)** Proposed mechanism linking the *MTHFR* C677T polymorphism to epigenetic, immune, and metabolic dysfunction in RSA. The TT genotype impairs MTHFR enzyme activity, elevating Hcy levels and impairing methyl group supply for epigenetic regulation. Concurrently, reduced enzymatic function downregulates CYP7A1, disrupting cholesterol metabolism and leading to aberrant bile acid synthesis. These combined epigenetic and metabolic alterations promote a Th17/Th1-skewed immune response in RSA patients carrying the *MTHFR* 677 TT genotype. Data are presented as mean ± SD. ∗*p* < 0.05, ∗∗*p* < 0.01, ∗∗∗*p* < 0.001, ns: not significant. Abbreviations: Hcy, Homocysteine; RSA, recurrent spontaneous abortion; *MTHFR*, methylenetetrahydrofolate reductase; SAM, S-adenosylmethionine; BA, bile acid; *MTHF*, methyltetrahydrofolate; E2, estradiol; T, testosterone; DMRs, Differentially Methylated Regions; KEGG, Kyoto Encyclopedia of Genes and Genomes.

Folate supplementation effectively lowered Hcy across all genotypes but did not significantly alter sex hormone levels (Fig. 1B, lower panel), clarifying the scope of folate's action. Immunologically, the *MTHFR* 677 TT group displayed significantly elevated Th17/Treg, Th17/Th2, and Th1/Th2 ratios (Fig. 1C), indicating a pro-inflammatory shift toward Th17 and Th1 responses. Consistently, a retrospective review revealed a higher historical burden of Type 2 immunity-related disorders (e.g., allergies) in *MTHFR* 677CC carriers, further contrasting their Th2-skewed profile against the Th17-skewed phenotype in the TT group (Fig. 1D).

To investigate genotype-specific metabolic perturbations, we performed quantitative targeted metabolomics on patients with *MTHFR* 677CC and 677 TT genotypes. OPLS-DA combined with univariate testing identified 21 differentially abundant metabolites spanning amino acid, fatty acid, bile acid, carbohydrate, carnitine, and organic acid pathways (Fig. S2). Given the established role of bile acids in Th17 cell differentiation, we compared the bile acid profiles between individuals carrying the *MTHFR* 677 TT genotype and those with the *MTHFR* 677CC/CT genotypes (Fig. S1). This analysis revealed reduced levels of both primary and secondary bile acids in *MTHFR* 677 TT carriers (Fig. 1E). Among the 41 bile acid metabolites quantified, integrated univariate and multidimensional analyses consistently identified significant alterations in 12 serum bile acid metabolites in the *MTHFR* 677 TT group (Table S2, Fig. 1F). Notably, with the exception of β-UDCA, all of these metabolites showed significantly lower concentrations in *MTHFR* 677 TT carriers. These findings link bile acid dysregulation to *MTHFR*-associated RSA and suggest a potential target for metabolic intervention.^2^

Given the critical role of *MTHFR* in one-carbon metabolism, we investigated the impact of the *MTHFR* C677T polymorphism on genome-wide methylation patterns in RSA patients.^3^ Whole-genome methylation sequencing revealed differentially methylated regions (DMRs) uniformly distributed across all chromosomes (Fig. 1G, left). Genome-wide methylation analysis revealed that hypomethylated DMRs outnumbered hypermethylated DMRs by 1.50-fold in *MTHFR* 677 TT carriers compared with CC carriers (Fig. 1G, middle, Fig. S3A). Furthermore, patients with the *MTHFR* 677 TT genotype showed significantly lower methylation levels at promoter-associated DMRs compared to the *MTHFR* 677CC carriers (Fig. 1G, right). In addition, promoter-associated DMRs were enriched in genes regulating Th17/Th2 differentiation and T-cell receptor signaling by Kyoto Encyclopedia of Genes and Genomes (KEGG) analysis (Table S3, Fig. 1H). Gene Ontology (GO) analysis further highlighted their involvement in cell surface receptor signaling and methyltransferase activity (Fig. S3B), providing an epigenetic basis for the observed immune shift.

We propose an integrated model wherein the *MTHFR* 677 TT genotype induces not only HHcy but also—through yet-undefined mechanisms—bile acid dysregulation and a DNA hypomethylation landscape. Hypomethylation may prime Th17/Th1-related genes for activation, while deficient bile acid signaling further disrupts immune homeostasis. Together, HHcy, hypomethylation, and bile acid suppression cooperatively drive a pathogenic Th17/Th1-skewed microenvironment, culminating in RSA with *MTHFR* 677 TT subgroup (Fig. 1I).^4^

These insights move beyond the classic “HHcy-centric” view and suggest combinatorial strategies: standard folate supplementation coupled with bile acid modulation or targeted Th17 pathway intervention may offer more effective management for *MTHFR* 677 TT-associated RSA.^5^

The *MTHFR* 677 TT genotype defines an RSA subgroup with a distinct multi-omics signature, integrating metabolic, immune, and epigenetic disturbances. Our study provides a systematic blueprint for understanding its pathogenesis and lays the scientific foundation for developing personalized therapeutic strategies.

## CRediT authorship contribution statement

**Na Yu:** Writing – review & editing, Writing – original draft, Data curation. **Yuxian Wu:** Writing – original draft, Visualization, Formal analysis. **Ting Sun:** Writing – review & editing. **Yang Liu:** Supervision, Methodology, Conceptualization. **Yaoyang Liu:** Writing – review & editing, Writing – original draft, Supervision, Conceptualization. **Shihua Bao:** Writing – review & editing, Supervision, Methodology.

## Ethics declaration

This study was approved by the Ethics Committee of Shanghai First Maternity and Infant Hospital (Approval number: KS23200) and was prospectively registered at the Chinese Clinical Trial Registry (ChiCTR197808). Written informed consent was obtained from all participants.

## Funding

This work was supported by the 10.13039/501100001809National Natural Science Foundation of China (No. 82071769), Shanghai Oriental Talent Youth Project (No. QNWS2024056), and Key Medical Specialties of Changning District Health Commission (No. 20231005).

## Conflict of interests

The authors declare that they have no competing interests.
