# Supplementary material for: Impact of MTHFR C677T polymorphism on recurrent spontaneous abortion: Bridging hyperhomocysteinemia, bile acid metabolism, and the epigenetic–immune axis
Source: Genes Dis. 2026 Jun 10;14(3):102293. doi: 10.1016/j.gendis.2026.102293 (PMC13742274; doi:10.1016/j.gendis.2026.102293)
Supplement: Supplementary file 1 [file mmc1.docx]

**Study subjects**

A cohort of 196 RSA patients was enrolled at Shanghai First Maternity and Infant Hospital between May 2023 and August 2024, with *MTHFR* C677T genotype distribution *MTHFR* 677CC/CT/TT = 66/64/66. Inclusion criteria comprised: (1) The diagnostic criteria for RSA were based on the Chinese Journal of Obstetrics and Gynecology, September 2022, Vol. 57, No. 9: 'Expert Consensus on the Diagnosis and Management of RSA(2022)'; (2) ≥2 pregnancy losses before 28 weeks' gestation (including biochemical pregnancy, empty gestational sac, spontaneous abortion, or embryonic arrest) with the same partner; (3) confirmed *MTHFR* C677T polymorphism; Exclusion criteria included: (1) no exposure to intravenous immunoglobulin, biologics, glucocorticoids (≥15 mg/day), or immunosuppressants within 3 months prior to enrollment; (2) uterine anomalies confirmed by ultrasonography; (3) chromosomal abnormalities in the products of conception or in either partner identified through karyotype analysis (Figure 1A).This study was approved by the Ethics Committee in the Hospital of Shanghai First Maternity and Infant Hospital (approval number KS23200)，prospectively registered at the Chinese Clinical Trial Registry (ChiCTR197808).

**Demographic and Clinical Data Collection**

Demographic data (age, obstetric history, infertility history) and clinical features (medication use, chronic comorbidities, miscarriages etiology and Type 2 immune related disorders) were systematically collected at enrollment, sourced from electronic medical records and/or structured questionnaires.

Laboratory Assessments

Laboratory analyses were conducted in the Department of Laboratory Diagnosis at Shanghai First Maternity and Infant Hospital. Peripheral blood samples were collected after an overnight fast and processed within 2 hours. Serum autoantibody profiles were quantified via enzyme-linked immunosorbent assay (ELISA), including: anti-cardiolipin antibodies (ACA-IgG/IgM), β₂-glycoprotein I antibodies (β₂GPI-IgG/IgM), anti-dsDNA, anti-Smith (Sm), anti-ribonucleoprotein (RNP), anti-SSA/Ro52, anti-SSB/La, anti-topoisomerase I (Scl70), anti-histidyl-tRNA synthetase (Jo-1), anti-thyroid peroxidase (TPO), anti-thyroglobulin (TG), anti-α-fodrin, anti-ssDNA, antinuclear antibodies (ANA), and extractable nuclear antigens (ENA). Lupus anticoagulant (LAC) was detected following ISTH guidelines using the dilute Russell’s viper venom time (dRVVT) and silica clotting time (SCT) assays (Sysmex, Japan), with results interpreted according to the manufacturer’s protocols. Coagulation parameters, including protein S (PS), protein C (PC), factor V (FV), antithrombin III (ATIII) activity, platelet aggregation (PAGT), and D-dimer (D-D) were evaluated using clotting-based assays on a Fully automatic coagulation analyzer (Sysmex, Japan). Hcy was analyzed via automatic biochemical analyser (Hitachi Labospect 008as, Japan). Hormonal profiles, including follicle-stimulating hormone (FSH), luteinizing hormone (LH), estradiol (E₂), prolactin (PRL), testosterone (T), dehydroepiandrosterone sulfate (DHEA-S), thyroid-stimulating hormone (TSH), free thyroxine (FT4), anti-Müllerian hormone (AMH), and insulin, were determined by electrochemiluminescence immunoassay on a Atellica Solution analyser (Siemens, Healthineers). 25-Hydroxyvitamin D (25(OH)D) and steroid hormones were measured by Architect i2000SR (Abbott, USA). Fasting glucose and lipid profiles (triglycerides, cholesterol) were analyzed enzymatically on a Hitachi Labospect 008 as autoanalyzer (Hitachi, Tokyo, Japan). Cytokine levels (IL-2, IL-4, IL-6, IL-10, TNF-α, IFN-γ, IL-17) were quantified via multiplex bead-based immunoassay (BD FACSCanto, USA) using a commercial kit (Jiangxi Cellgene Biotech Co, P110100203, China). Immunoglobulins (IgA, IgM, IgG) and ferritin were measured by nephelometry on an Atellica NEPH 630 (Siemens, Germany). Complete blood counts (leukocytes, lymphocytes, hemoglobin, platelets) and inflammatory markers (C-reactive protein, erythrocyte sedimentation rate) were evaluated by immunoturbidimetry and Westergren method, respectively, on a COBAS 8000 system (Roche Diagnostics). *MTHFR* C677T gene polymorphism was detected by a polymerase chain reaction and reverse hybridization using the *MTHFR* 677CT RealFastTM Assay (ViennaLab Diagnostics GmbH, Vienna, Austria) according to the manufacturer’s instructions.

**Sample collection**

Peripheral blood samples were collected from fasting participants and processed within 2 hours. Whole blood aliquots for flow cytometry were maintained at 4°C and analyzed within 24 hours of collection. Remaining samples underwent centrifugation at 3000g for 15 min at 4°C to separate plasma and cellular components. Plasma was aliquoted for metabolomic profiling, while blood cells were preserved for methylation analysis. All aliquots were snap-frozen in liquid nitrogen and stored at -80°C until subsequent analysis (metabolomics by ultra-performance liquid chromatography-tandem mass spectrometry [UPLC-MS/MS] and DNA methylation by reduced representation bisulfite sequencing [RRBS]).

**Antibodies**

CD45 (mFluor540, Caprico, S045MF24), HLA-DR (BV421, BD Biosciences, 664141), CD8 (APC-CY7, 4A BIOTECH, 008-109-3), CD4 (Alexa Fluor 700, Biolegend, 344622), CD3 (APC, BD Biosciences, 665333), TCRγδ (PE-Cy7, Beckman Coulter, B10247), CD196 (PE-Cy7, Biolegend, 353418), CD127 (PC5, Beckman Coulter, A64617), CD45RA (PC5, Beckman Coulter, B49193), CD25 (PE, Beckman Coulter, A07774) and CD183 (FITC, Biolegend, 353710)

**Flow Cytometry**

Peripheral blood samples (100 μL) were collected and stained with the following fluorescently-labeled anti-human antibodies: CD45, HLA-DR, CD8, CD4, CD3, TCRγδ, CD196 (CCR6), CD127 (IL-7Rα), CD45RA, CD25 (IL-2Rα), and CD183 (CXCR3). The staining mixture was incubated for 15 min at room temperature in the dark. Following surface staining, red blood cells were lysed using BD FACS™ Lysing Solution according to the manufacturer's protocol. Samples were analyzed on a Beckman Coulter DxFLEX flow cytometer. Data analysis was performed using Kaluza Analysis Software v2.1, with appropriate gating strategies applied to identify specific lymphocyte populations. The immune cell subsets were defined as follows: Th1: CD45^+^CD3^+^CD4^+^CD183^-^CD196^+^, Th2: CD45^+^CD3^+^CD4^+^CD183^-^CD196^-^, Th17: CD45^+^CD3^+^CD4^+^CD183^-^CD196^+^, Treg: CD45^+^CD3^+^CD4^+^CD127^dim^CD25^high^, Active Treg:CD45^+^CD3^+^CD4^+^CD127^lo/-^CD25^+^HLA-DR^+^, Naive Treg: CD45^+^CD3^+^CD4^+^CD127^lo/-^CD25^+^CD45RA^+^, Effective Memory Treg: CD45^+^CD3^+^CD4^+^CD127^lo/-^CD25^+^CD45RA^-^, CD8^+^T cells: CD45^+^CD3^+^CD8^+^, γ/δT cells:CD45^+^CD3^+^CD4-CD8-TCRγδ^+^.

**Metabolomic profiling**

Frozen plasma samples were thawed on ice, added with ice-cold methanol containing internal standard (IS), then vortexed and centrifuged. The supernatant was collected, mixed with derivatization reagent, and incubated. After dilution, the sample was re-centrifuged, and the resulting supernatant was added with IS and derivatization standard solution. Metabolite quantification was performed using ultra-performance liquid chromatography-tandem mass spectrometry (UPLC-MS/MS) (ACQUITY UPLC Xevo TQ-S, Waters Corp., Milford, MA, USA). Separation was achieved using an ACQUITY UPLC BEH C18 VanGuard pre-column (2.1 × 5 mm, 1.7 μm) and an ACQUITY UPLC BEH C18 analytical column (2.1 × 100 mm, 1.7 μm). The mobile phase consisted of (A) water with 0.1% formic acid and (B) a mixture of acetonitrile and isopropanol (70:30, v/v). The gradient elution program was as follows: 0–1 min (5% B), 1–11 min (5–78% B), 11–13.5 min (78–95% B), 13.5–14 min (95–100% B), 14–16 min (100% B), 16–16.1 min (100–5% B), and 16.1–18 min (5% B). Various classes of metabolites, including bile acids, fatty acids, and amino acids, were analyzed in multiple reaction monitoring (MRM) mode.

****Bile acid analysis****

Plasma samples were mixed with ice-cold acetonitrile/methanol (8:2, v/v) containing isotopically labeled internal standards. Following vertexing, samples were centrifuged, and the supernatants were collected and lyophilized using a freeze dryer (Labconco, Kansas City, MO, USA). The dried samples were reconstituted in acetonitrile/methanol solution (80:20, v/v) along with calibration standards and quality control samples, followed by vortexing. Deionized water was then added, and the mixture was further vortexed at 10°C. Proteins were precipitated by incubating the mixture at -20°C, followed by centrifugation prior to UPLC-MS/MS analysis. Chromatographic separation was carried out on an ACQUITY UPLC system (Waters Corp., Milford, MA, USA) using a Cortecs C18 VanGuard pre-column (2.1 × 5 mm, 1.6 μm) and an ACQUITY UPLC Cortecs C18 analytical column (2.1 × 100 mm, 1.6 μm). The mobile phase consisted of (A) 10 mM ammonium acetate with 0.25% acetic acid and (B) a mixture of acetonitrile/methanol/isopropanol (8:1:1, v/v/v). The gradient elution program was as follows: 0–0.3 min (5% B), 0.3–0.5 min (5–10% B), 0.5–2 min (10–15% B), 2–3 min (15–30% B), 3–6 min (30% B), 6–8 min (30–35% B), 8–9 min (35–40% B), 9–10 min (40% B), 10–15 min (40–75% B), 15–15.5 min (75–100% B), 15.5–16.2 min (100% B), 16.2–16.3 min (100–5% B), and 16.3–17 min (5% B).

**Metabolomic Data Analysis**

Data acquisition and processing were performed using TMBQ software (v1.0, Metabo Profile, Shanghai, China), MassLynx software (v4.1, Waters, Milford, MA, USA), and the iMAP platform (v1.0, Metabo Profile, Shanghai, China). Multivariate analysis was conducted via orthogonal partial least squares discriminant analysis (OPLS-DA), and metabolites with variable importance in the projection (VIP) scores >1.0 were considered discriminative for classification. The OPLS-DA quality control plot is presented in Supplementary Figure S1A and B. Univariate tests included Student’s t-test (for normally distributed data) or Mann-Whitney U test (for nonparametric data). Differentially abundant metabolites were defined as those meeting both VIP >1.0 and univariate significance (*p* < 0.05). Prior to visualization, the final data were log2-transformed and normalized to Z-scores (mean = 0, standard deviation = 1). The differential metabolites identified by comprehensive quantitative metabolomics were presented in Supplementary Figure S2, while those identified by bile acid metabolomics were shown in Figure.1F and Supplementary Table 2.

**DNA Isolation**

Extraction of DNA using magnetic bead method (TIANGEN, DP705). Homogenize blood clots in cell lysis buffer (CLA) to dissociate visible clots, followed by centrifugation to isolate the nuclear pellet. Resuspend the pellet in CLA buffer and centrifuge to remove residual contaminants. Digest proteins using Proteinase K and GHL lysis buffer at 75°C. Transfer the supernatant, precipitate DNA with isopropanol, and bind to magnetic beads. Purify DNA using magnetic separation.

**Reduced Representation Bisulfite Sequencing (RRBS)**

Following DNA quality assessment, a 0.1% spike-in of lambda DNA (methylation-negative control) was added. Genomic DNA underwent digestion with MspI (methylation-insensitive restriction enzyme), followed by end repair, 3'-adenylation, and ligation to methylated adapters. Fragments of 40-220 bp were size-selected by agarose gel electrophoresis, then bisulfite-converted using the EZ DNA Methylation-Gold™ Kit (Zymo Research, Cat# D5005), which deaminates unmethylated cytosines to uracils (PCR-converted to thymines) while preserving methylated cytosines. Final libraries were PCR-amplified, quality-controlled (Qubit dsDNA HS Assay; Agilent 2100 Bioanalyzer), and sequenced on Illumina NovaSeq X Plus (150 bp PE).

**RRBS Data Analysis**

Raw data quality was assessed using FastQC (v0.11.8). Sequencing reads in FASTQ format from the Illumina platform were preprocessed with fastp (v0.23.1) to generate clean data, which were re-evaluated with FastQC and used for downstream analyses. Bisulfite-treated reads were aligned to the reference genome using Bismark (v0.24.0) with parameters -X 700 -dovetail. Methylation analysis was visualized using IGV. Methylated cytosines were identified via binomial tests using methylated C counts (mC), total C counts (mC+umC), and non-conversion rates (r), with FDR-corrected *p*<0.05 and a minimum sequencing depth of ≥15× per sample. Differentially methylated regions (DMRs) were analyzed using the DSS package. Supplementary Figure S3 A depicts a heatmap comparing DNA methylation levels at DMRs in RSA patients with the *MTHFR* 677TT versus the CC genotype. The detailed information on the DMRs in promoter regions is provided in Supplementary Table 3.

GO (Gene Ontology) enrichment analysis of DMR-associated genes was performed using the R package GOseq, with significance set at *p*<0.05. Length bias-corrected GO enrichment was determined using GOseq, considering Corrected_*p* value <0.05 as significant. KEGG pathway enrichment analysis was conducted using KOBAS software.

**Statistical analysis**

Continuous variables were presented as median (interquartile range, IQR). Categorical variables were expressed as frequency (percentage). Continuous variables were tested for normality, and nonparametric Mann-Whitney U tests were used for two-group comparisons due to their predominantly non-normal distribution. Categorical variables were analyzed via chi-square tests. All statistical analyses applied a two-tailed significance level of *p* < 0.05 and were performed using SPSS Statistics (Version 29.0.2.0). Data visualization was performed using GraphPad Prism (Version 10.1.2).

**Supplementary Figures**

Supplementary Figure S1. Quality control analysis of metabolomics and methyl omics data stratified by MTHFR genotype. (A) OPLS-DA score plots of the metabolomics data for RSA patients with *MTHFR* 677TT and CC/CT genotypes. (B) Permutations in RSA patients with *MTHFR* 677TT and CC/CT genotypes. Abbreviations: *MTHFR*: methylenetetrahydrofolate reductase; RSA: recurrent spontaneous abortion; OPLS-DA: Orthogonal Partial Least Squares Discriminant Analysis.

Supplementary Figure S2. Metabolomic profiling in RSA patients. Heatmap displays differentially abundant metabolites identified through univariate and multivariate analyses, encompassing primary and secondary bile acids, amino acids, carbohydrates, carnitines, fatty acids, and organic acids. Abbreviations: *MTHFR*: methylenetetrahydrofolate reductase; RSA: recurrent spontaneous abortion.

Supplementary Figure S3. DNA methylation analysis in RSA patients. (A) Heatmap visualization of differential DNA methylation profiles in RSA patients stratified by *MTHFR* C677T genotype (TT vs. CC). (B) GO enrichment analysis for all differentially methylated CpG sites. Bar chart shows functional categorization of genes based on biological processes, cellular components, and molecular functions. The x-axis indicates gene counts, the y-axis shows specific GO terms. Abbreviations: *MTHFR*: methylenetetrahydrofolate reductase; RSA: recurrent spontaneous abortion; DMRs: differentially methylated regions; GO: Gene Ontology

Supplementary Figure S4. Auto-antibody profiles in RSA patients stratified by MTHFR C677T genotype (TT vs. CT vs. CC). Abbreviations: *MTHFR*: methylenetetrahydrofolate reductase; RSA: recurrent spontaneous abortion.

**Supplementary Tables**

Supplementary Table.1.1 Baseline Traits of *MTHFR* 677TT vs. *MTHFR* 677CC/CT Genotypes in enrolled RSA Patients.

Supplementary Table.1.2 Comparative Analysis of Metabolic Characteristics in RSA Patients with *MTHFR* 677TT vs. *MTHFR* 677CC/CT Genotypes.

Supplementary Table.1.3 Comparative Analysis of Hormone Level in RSA Patients with *MTHFR* 677TT vs. *MTHFR* 677CC/CT Genotypes.

Supplementary Table.1.4 Comparative Analysis of Hematological, Inflammatory, and Coagulation Profiles in RSA Patients with *MTHFR* 677TT vs. *MTHFR* 677CC/CT Genotypes.

Supplementary Table.1.5 Comparative Analysis of Peripheral Blood T Lymphocyte Subsets in RSA Patients with *MTHFR* 677TT vs. *MTHFR* 677CC/CT Genotypes.

Supplementary Table.1.6 Comparative Analysis of Immunological Characteristics in RSA Patients with *MTHFR* 677TT vs. *MTHFR* 677CC/CT Genotypes.

Supplementary Table.1.7 Comparative Analysis of Autoantibody Profiles in RSA Patients with *MTHFR* 677TT vs. *MTHFR* 677CC/CT Genotypes.

Supplementary Table.2 Comparison of Bile Acid Metabolite Concentrations in RSA Patients with *MTHFR* 677TT vs. *MTHFR* 677CC/CT Genotypes.

Supplementary Table.3 Comparison of Promoter Methylation Levels in RSA Patients with *MTHFR* 677TT vs. *MTHFR* 677CC Genotypes.
